# Comprehensive Overview of Antibacterial Drugs and Natural Antibacterial Compounds Found in Food Plants

**DOI:** 10.3390/antibiotics14020185

**Published:** 2025-02-11

**Authors:** Sabine Berteina-Raboin

**Affiliations:** Institut de Chimie Organique et Analytique (ICOA), Université d’Orléans, UMR-CNRS 7311, BP 6759, Rue de Chartres, CEDEX 2, 45067 Orleans, France; sabine.berteina-raboin@univ-orleans.fr; Tel.: +33-238494856

**Keywords:** antibiotics classes, natural compounds, resistance, activity

## Abstract

The aim of this review is to list the various natural sources of antimicrobials that are readily available. Indeed, many plant sources are known to have antibiotic properties, although it is not always clear which molecule is responsible for this activity. Many food supplements also have this therapeutic indication. We propose here to take stock of the scientific knowledge attesting or not to these indications for some food sources. An overview of the various antibiotic drugs commercially available will be provided. A structural indication of the natural molecules present in various plants and reported to contribute to their antibiotic power will be given. The plants mentioned in this review, which does not claim to be exhaustive, are referenced for fighting Gram-positive and/or Gram-negative bacteria. It is difficult to attribute activity to just one of these natural molecules, as it is likely to result from synergy within the plant. Similarly, chitosan is mentioned for its fungistatic and bacteriostatic properties. In this case, this polymeric compound derived from the chitin of marine organisms is referenced for its antibiofilm activity. It seems that, in the face of growing antibiotic resistance, it makes sense to keep high-performance synthetic antibiotics on hand to treat the difficult pathologies that require them. On the other hand, for minor infections, the use of better-tolerated natural sources is certainly sufficient. To achieve this, we need to take stock of common plant sources, available as food products or dietary supplements, which are known to be active in this field.

## 1. Introduction

After several epidemics exacerbated by poor hygiene, overpopulation, and difficult living conditions, the prevalence of infectious diseases has been declining for several decades. This is due to the industrial development of a range of high-performance, targeted, broad-spectrum antibiotics. Unfortunately, their intensive use in human and animal health, combined with poor compliance with medical recommendations, has led to the emergence of resistance, which continues to grow [1,2,3,4,5]. This multi-resistance is inescapable, given the natural evolution of bacteria, and is extremely worrying. Indeed, aging populations are accompanied by an increase in the number of immunocompromised people, who are more susceptible to bacterial attack. All this is contributing to the resurgence of infectious diseases that were thought to have been eradicated, such as tuberculosis. Hospitals are also witnessing an increase in bacterial infections because of more and more extensive surgery and cancer therapies. This extensive use is conducive to resistance [6]. In addition, the development of new antibiotics has fallen off sharply since the end of the last century. This situation raises medical concerns about our future ability to overcome these resistance problems. They are due to a number of factors, both in primary care (ambulatory medicine) and in hospital medicine. Resistance began with Gram-negative (double-membrane) bacteria, notably in the 1950s with sulfonamides against *N. meningitidis*. In the same years, penicillin resistance began in Gram-positive bacteria such as *S. aureus*. Today, more and more bacteria are becoming resistant to the various classes of antibiotics, and it only takes a few years for bacteria to adapt and develop defense mechanisms that are part of natural evolution [4,5]. Given the global scale of infectious diseases, the World Health Organization considers antibiotic resistance to be a major public health problem [7]. Bacteria are not the only ones to resist drugs: viruses, fungi, and parasites can also adapt. Research is therefore focusing on ways to prevent infections by limiting the transmission of bacteria, but also the development of resistance genes. The medical world is also focusing on improving recommendations for the use of antibiotics. Indeed, it has now been proven that incorrect application of recommendations in terms of antibiotic quantities and days of administration has a major impact on treatment efficacy. Antibiotics used incorrectly encourage antibiotic resistance in target bacteria [8]. There are two types of resistance: either natural resistance (hence the need for several classes of antibiotics) or acquired resistance. This one may result from a chromosomal mutation, which is rather rare, or from the acquisition of genetic material such as plasmids, which is more frequent. This resistance can result in permeability problems for antibiotics, either limiting their penetration or ejecting them rapidly. It may also involve enzymes capable of destroying antibiotics. On the other hand, natural antibiotics traditionally used to treat minor pathologies do not seem to suffer from a drop in activity [9]. They are therefore not subject to the same types of resistance. There may be many reasons for this. These natural molecules are commonly ingested and they form part of our dietary arsenal. Their use in food supplements could limit the use of antibiotics, which might be more effective but not necessarily required for the pathology in question. This first-line use could limit the use of antibiotics, preserving their efficacy for more severe infections. Similarly, feeding animals’ forages containing natural antibiotics could limit the use of synthetic molecules in animal health. It should be noted that, as with other drugs [10], some of these non-metabolized human or animal health molecules as well as their metabolites can end up in wastewater or runoff, and thus in the environment. These discharges, through their contamination of the natural environment, play a major role in antibiotic resistance. It is therefore essential to limit the use of antibiotics, and especially their misuse. It is essential to consider solutions for environmental protection and water treatment. We also need to master the biological mechanisms of resistance in order to limit them and preserve our ability to treat. In this review, we will take stock of the different families of synthetic or semisynthetic antibiotics, and look at the most commonly recognized so-called natural antibiotics, contained in plant foods and herbs. Many of these plant sources contain molecules with antibacterial potential and a broad spectrum of activity. These include phenol derivatives, aromatic aldehydes, and terpene alcohols. We will provide information on their composition, with a view to exploring new routes of research for the discovery of new molecular targets. Indeed, the antibiotics that remain active on multi-resistant bacteria are often those that present an increased risk of side effects, and therefore potentially limit their use. In general, they are mainly prescribed in hospital settings to guarantee these higher risks, which does not facilitate patient management.

The first commercially available antibiotics of the first half of the last century were sulfonamides followed by penicillin (or more commonly β-lactams), met with so-called natural resistance from the outset. Some antibiotics target a narrow range of bacteria while other molecules are said to be broad-spectrum because they have a wider bacterial panel, in addition to the aforementioned adaptation of bacteria, like all living organisms, to the environmental hazards of antibiotics. This rapid adaptation is also due to the increasing dissemination of bacteria that have become resistant to them.

This multi-resistance and closely related cross-transmission lead to serious difficulties in the therapeutic treatment of infections of all types. Fortunately, infections caused by opportunistic bacteria such as staphylococci, enterobacteria, non-tuberculous mycobacteria, listeria, bacillus, etc., do not appear to be cross-transmitted. However, multidrug resistance is a major cause of death, particularly in hospitals [2,11,12].

## 2. Different Classes of Compounds with Antibacterial Activity

There are eight main classes or chemical families of synthetic antibiotics that inhibit the growth of microorganisms such as bacteria and other microbes [11,12,13]. These are small molecules designed to be well tolerated, yet effective against the targeted micro-organisms. They have their own spectrum of activity and contraindications. The different molecules diffuse differently in the various organs and/or body fluids, have different therapeutic targets, and different recommendations in terms of doses and duration of treatment.

### 2.1. β-Lactams

**Figure 1:**
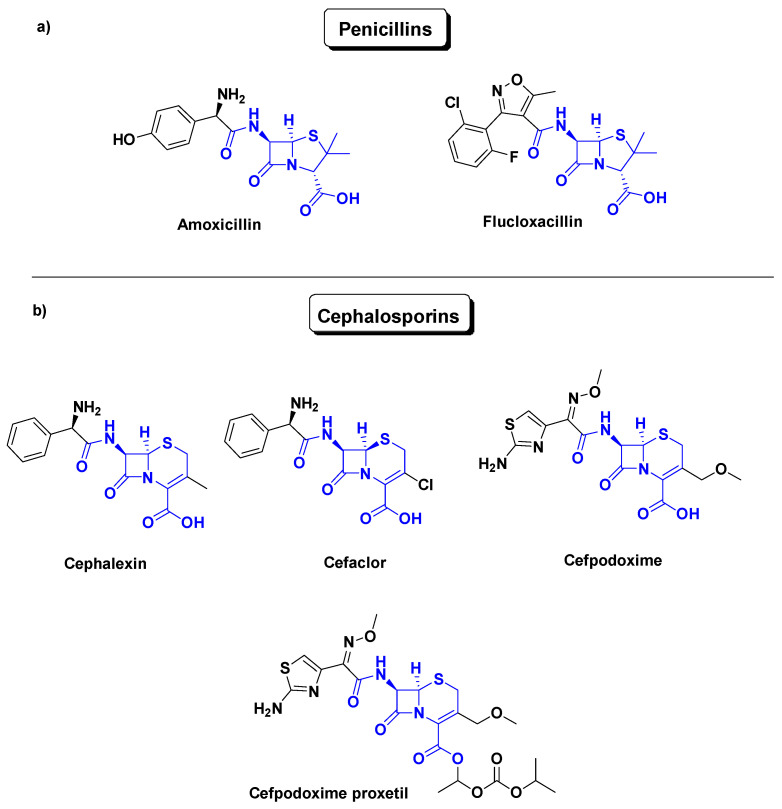
Common β-Lactams, (**a**) penicillins, and (**b**) cephalosporins.

The oldest family is the β-lactam family, which inhibits bacterial peptidoglycan cell wall synthesis [14]. This family acts as bactericidal agent and includes penicillin (amoxicillin, flucloxallin) and cephalosporins (cephalexin, cefaclor, cefpodoxime, cefpodoxime proxetil), which have a similar mode of action and are commonly prescribed, but remain narrow-spectrum antibiotics. Amoxicillin, flucloxallin, cephalexin (first-generation,) cefaclor (second-generation), cefpodoxime (third-generation) and its ester prodrug cefpodoxime proxetil are semisynthetic compounds. They target Gram-positive bacteria such as *Staphylococcus*, *Streptococcus*, and *Gonococcus*. These antibiotics, as shown in Figure 1, consist of a cyclic amide. This constrained lactam is quite sensitive to hydrolysis, whether chemical [15] or enzymatic (β-lactamase) [16].

#### 2.1.1. Monobactams

**Figure 2:**
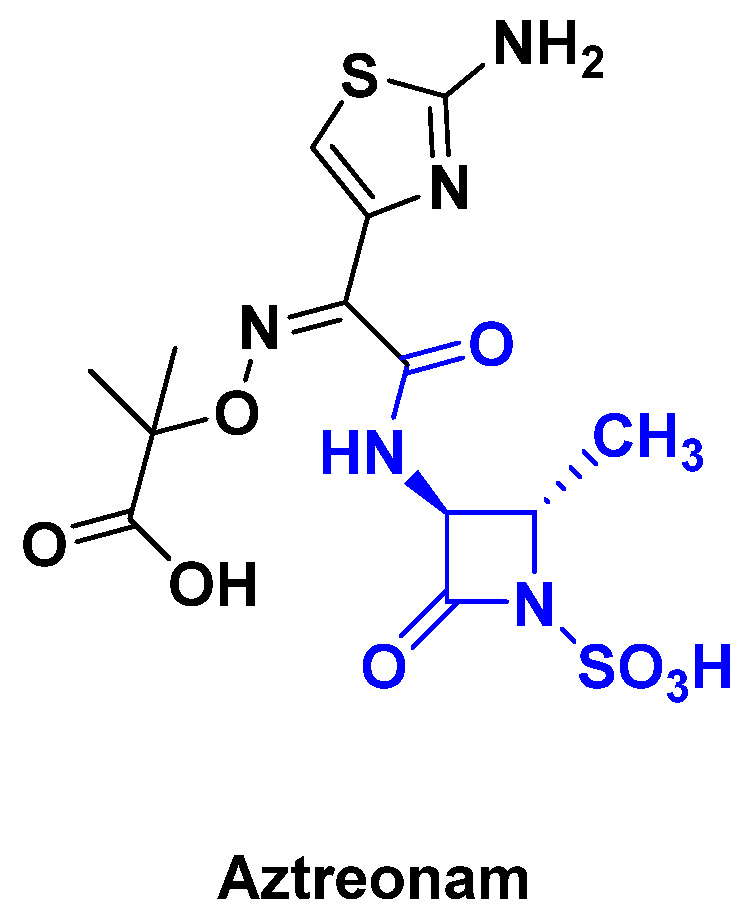
Chemical structure of monocyclic β-lactamine: Aztreonam.

Monobactams consist of a non-fused β-lactam ring. Aztreonam, a marketed monobactam antibiotic, is not active on Gram-positive bacteria, but only on certain Gram-negative bacteria, and has a fairly narrow spectrum of action (Figure 2) [17].

#### 2.1.2. Carbapenems

**Figure 3:**
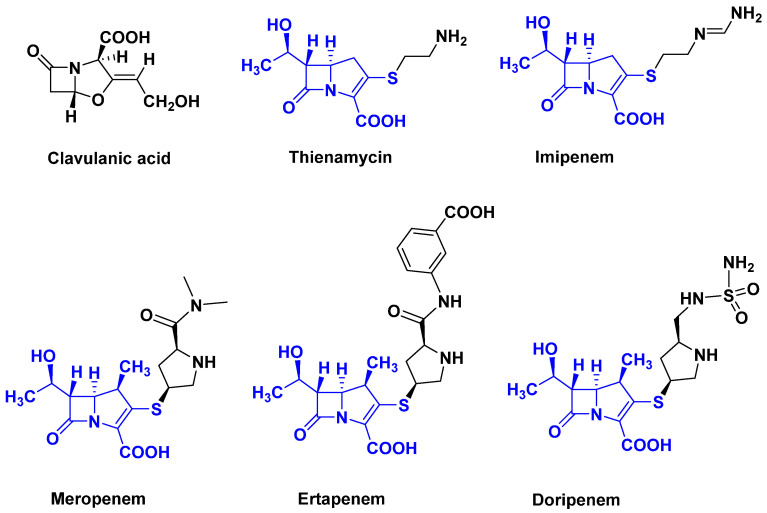
Clavulanic acid, thienamycin, and some carbapenems.

With the emergence of bacterial β-lactamases generating resistance to penicillin [18], the search for β-lactamase inhibitors led to the discovery of two compounds: clavulanic acid [19] and thienamycin [20]: the first carbapenem [18], followed by many others. Carbapenems discovered in the 1970s [21] are broad-spectrum antibiotics and are extremely potent against both Gram-positive and Gram-negative bacteria [22]. Carbapenems differ from penicillin by the presence of a carbon atom in place of the sulfur in position 1. Like cephalosporins, carbapenems have an unsaturated bond in C2–C3. This family includes the following drugs: Ertapenem used for many infections; Doripenem mainly capable of killing *P. aeruginosa*; Imiprenem mainly capable of killing *P. aeruginosa* and various *Entercoccus*; Meropenem used against *Pseudomonas* and *Enterobacteriaceae*; the list of drugs and bacteria targeted is far from exhaustive (Figure 3).

### 2.2. Sulfonamides

**Figure 4:**
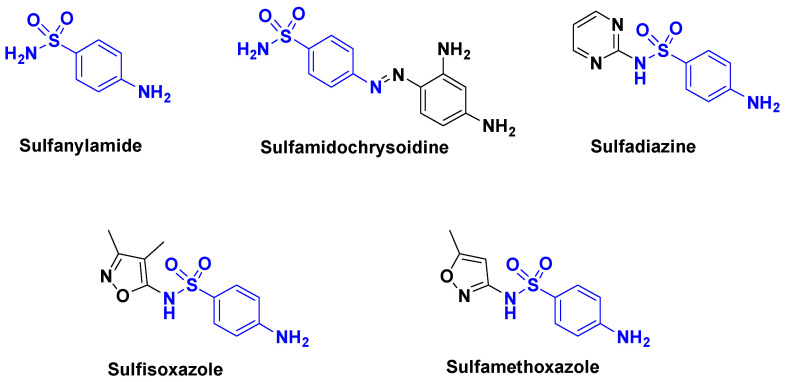
Some sulfonamides compounds.

Sulfonamides are bacteriostatic agents that limit growth and reproduction by inhibiting part of the bacterial synthesis of folic acid. They thus block the biosynthesis of purines, pyrimidines, and ultimately bacterial DNA in all living cells, which requires the presence of folates. However, they can have allergic side effects. This family includes the following drugs: sulfanilamide, sulfamidochrysoidine, sulfadiazine, sulfisoxazole, sulfamethoxazole. Like many drugs, sulfonamides are not fully metabolized and are found in wastewater [23]. In particular, sulfamethoxazole is frequently found (Figure 4).

### 2.3. Aminoglycosides

**Figure 5:**
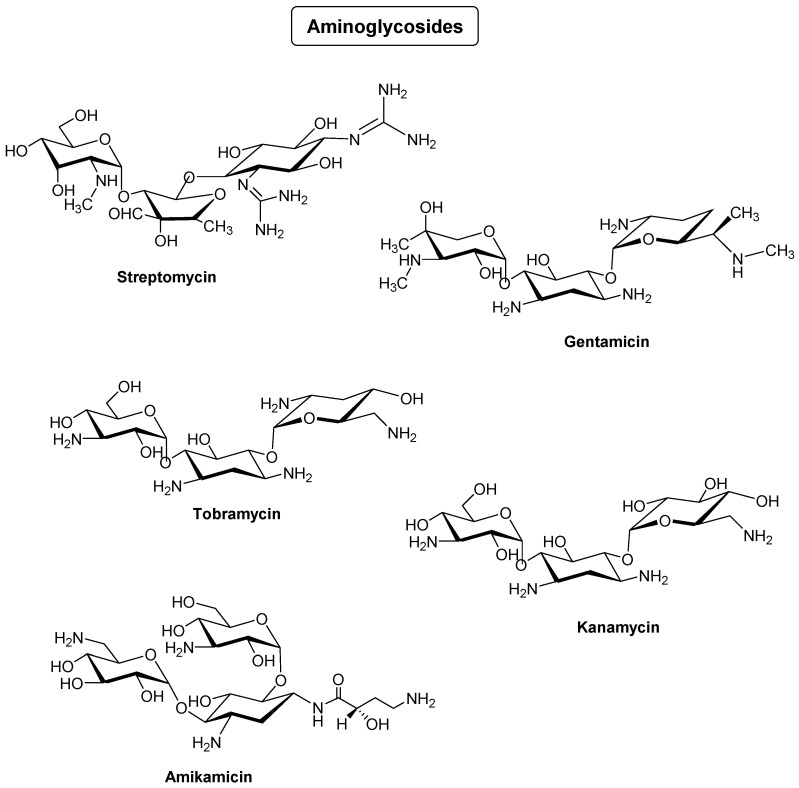
Some examples of aminoglycosides used as antibiotics.

Aminoglycosides consist of amino sugars linked to an aglycone: they are broad-spectrum antibiotics that inhibit protein synthesis in bacteria [24]. Streptomycin is the first drug in this family used to fight against tuberculosis in the elderly [25,26]. Unfortunately, this compound has proved toxic [27], and various analogs gentamycin, tobramycin, kanamycin, and amikacin have been developed and used. They act as bactericidal agents and are used in hospitals for the treatment of serious human infections caused by Gram-negative but also Gram-positive bacteria [28]. These antibiotics can also be used in animal health. As mentioned above, their antimicrobial action is based on inhibition of protein synthesis leading to cell death. They are often used in injectable form and mainly in hospitals, due to their significant side effects. Like sulfonamides, aminoglycosides are poorly metabolized and end up in wastewater. This problem can also lead to an increase in antibiotic resistance (Figure 5).

### 2.4. Tetracyclines

**Figure 6:**
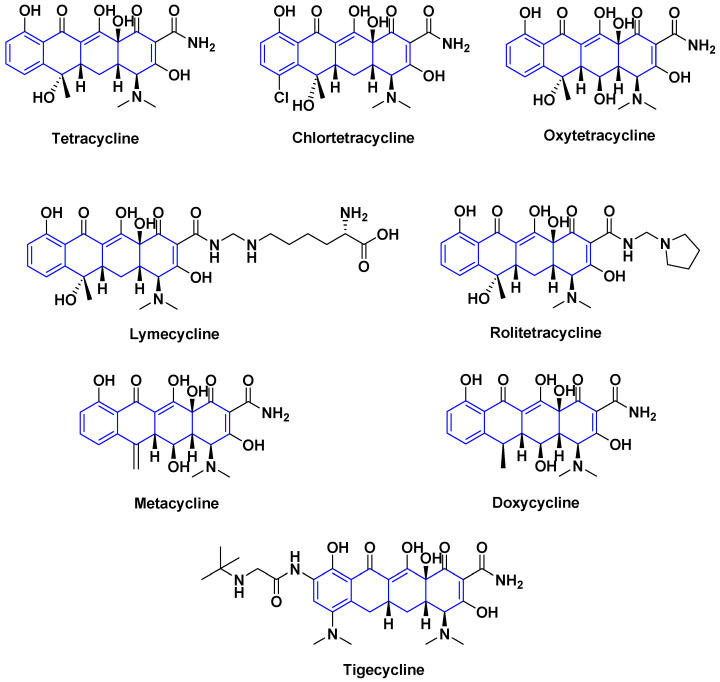
Various tetracyclines antibiotics.

Tetracycline is a broad-spectrum antibiotic discovered in 1945 and effective against a wide range of bacterial strains [29]. It consists of four fused rings, variously substituted. This bacteriostatic agent inhibits protein synthesis and limits bacterial growth [30]. However, it is much less widely used, even though it was highly effective against cholera, for example, as it was subject to resistance resulting from changes in microbial cell permeability that no longer allow adequate transport of the drug [31]. Available derivatives including tetracycline, chlortetracycline, and oxytetracycline are biosynthetic compounds, while metacycline, precursor in synthesis of doxycycline, lymecycline more soluble, rolitetracycline, and tigecycline [32] are obtained hemi-synthetically or synthetically, but the list is not exhaustive (Figure 6).

### 2.5. Macrolides

**Figure 7:**
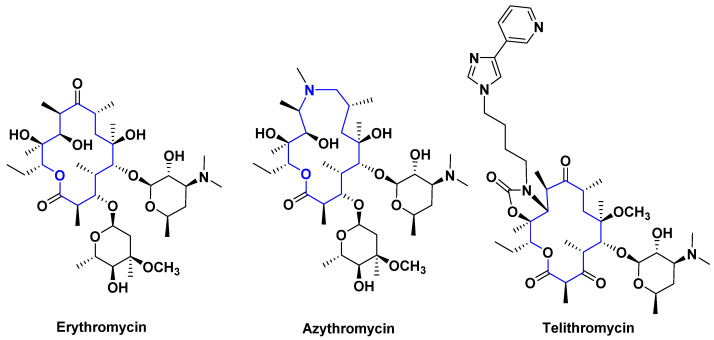
Some examples of macrolides.

Macrolides are some of the most widely prescribed antibiotics in the natural polyketide class. They consist of a 14- to 16-membered lactone to which are attached modified sugars such as cladinose and/or desosamine. One of the best-known macrolide antibiotics is erythromycin but telithromycin and azithromycin also belong to this group (Figure 7). As bacteriostatic agents, they inhibit bacterial protein synthesis, leading to bacterial death [33]. Their spectrum of antibiotic activity is broader than that of penicillin, hence their frequent use for patients who are allergic to penicillin [4,34]. Unfortunately, these macrolides are also widely excreted in wastewater without modification [35], which again contributes to resistance phenomena.

#### 2.5.1. Ansamycins

**Figure 8:**
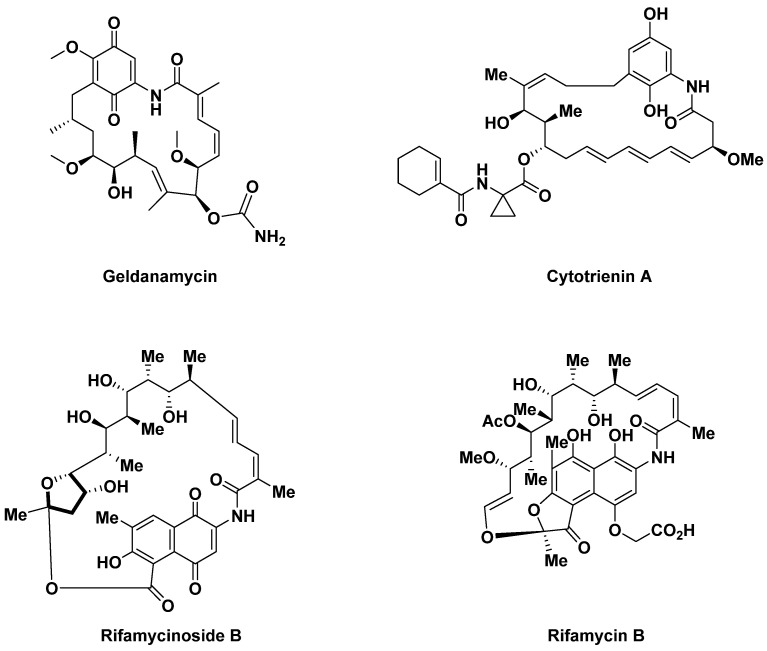
Examples of ansamycins.

Benzoquinone-type ansamycins in C15 (geldanamycin) or C17 (cytotrienin A) have a wide range of activities. Naphthalene-type ansamycins in C15 (rifamycininoside B) or C17 (rifamycin B) are molecules that block transcription and RNA polymerization. These molecules are also mainly used in oncology [36] (Figure 8).

#### 2.5.2. Lincosamides and Streptogramines

**Figure 9:**
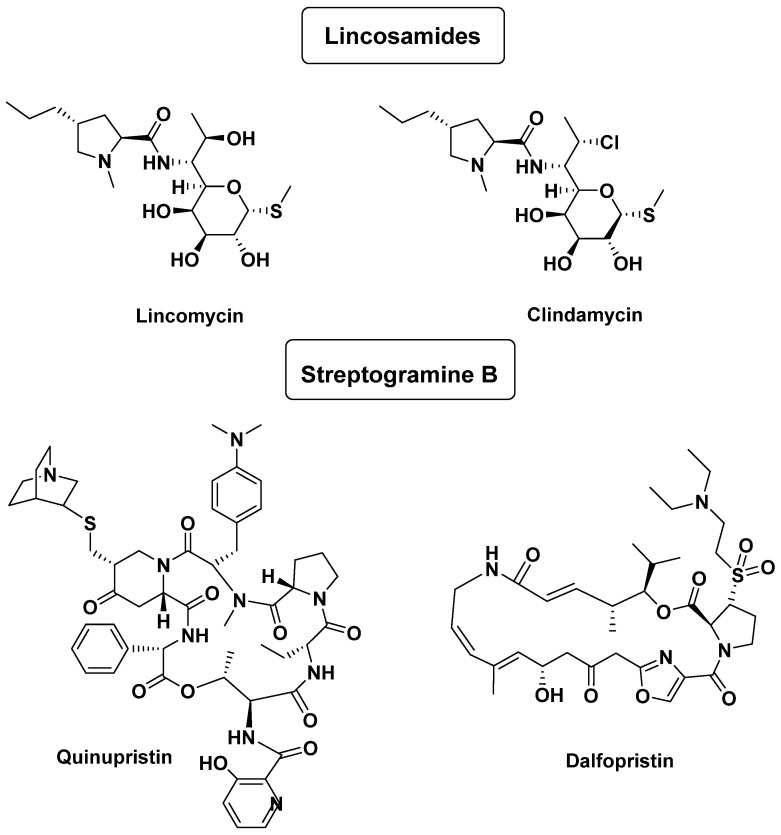
Examples of lincosamides and streptogramine B.

Lincosamides, lincomycin, and clindamycin were used to act against some methicillin-resistant *S. aureus* (MRSA), as well as streptogramines, for example, pristinamycin, that is a mixture of two compounds; it is the same thing for quinupristin-dalfopristin (Figure 9), which belongs to the macrolide class. These antibiotics have a very narrow spectrum of activity, similar to erythromycin. They are bactericidal agents, causing cell death by inhibiting protein synthesis [37].

### 2.6. Oxazolidinones

**Figure 10:**
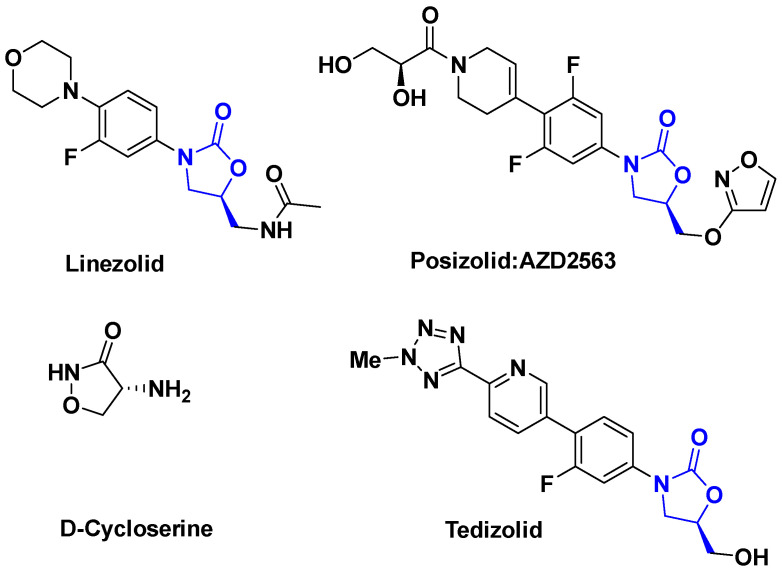
Some examples of oxazolidinones antibiotics.

Oxazolidinones are among the synthetic antibiotics, which are quite potent and bacteriostatic. They have a broad spectrum of activity, inhibiting the synthesis of proteins that limit bacterial growth. They are active against Gram-positive bacteria and are still effective on bacteria that have shown resistance to other antibiotics. Molecules in this class include the first to be synthesized, linezolid, used against some MRSA as well as posizolid, and tedizolid, used against methicillin and vancomycin-resistant bacteria to which should be added the simple D-cycloserine (Figure 10) [38].

### 2.7. Glycopeptides

**Figure 11:**
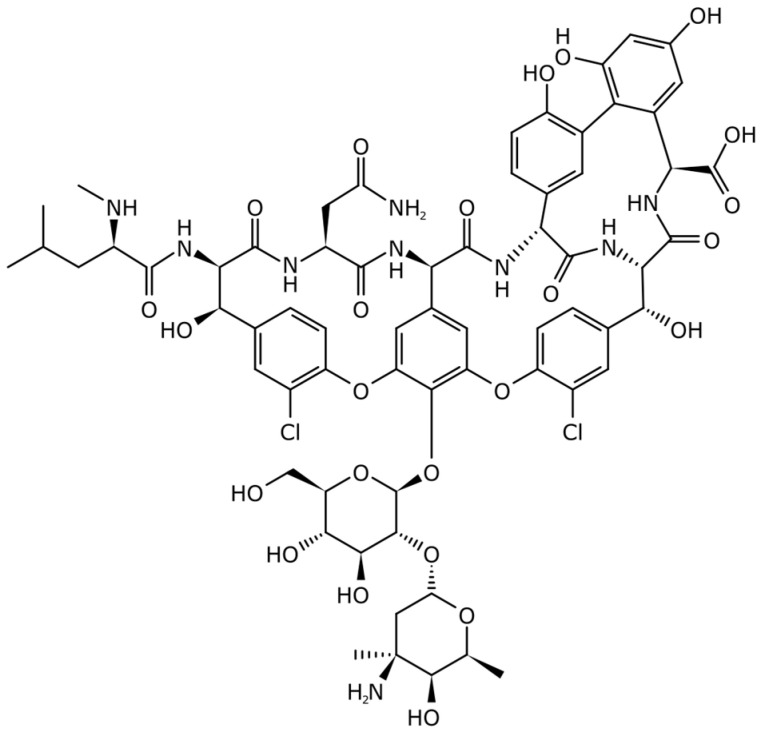
Example of glycopeptide: vancomycin.

Glycopeptide antibiotics are either natural or semisynthetic. They consist of a cyclic peptide with two sugar units as vancomycin [39,40]. Some molecules are pharmacomodulated to enhance their antibacterial potency, with a halogen atom in place of a hydrogen or an additional sugar, such as oritavancin [41,42] or even the addition of lipophilic side chains (Figure 11).

#### Lipoglycopeptides and Lipopeptides

**Figure 12:**
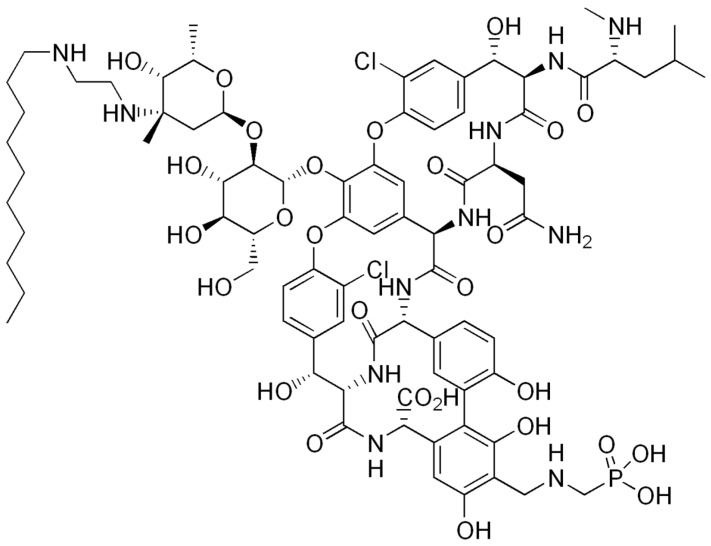
Example of lipoglycopeptide: telavancin.

**Figure 13:**
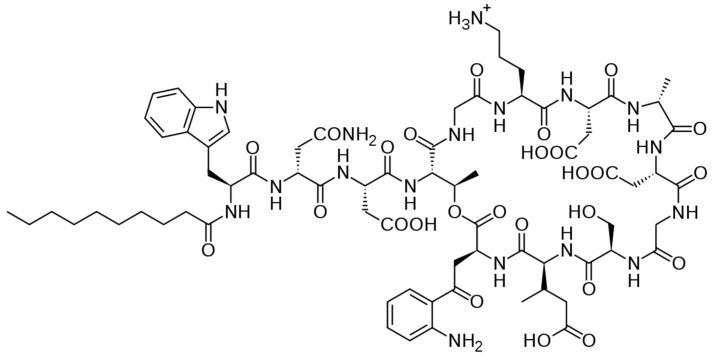
Example of lipopeptide: daptomycin.

Some semisynthetic lipoglycopeptide derivatives were subsequently developed, such as telavancin (Figure 12), dalbavancin, and oritavancin for dermatological use. These derivatives have a broader spectrum of activity than glycopeptides on Gram-positive bacteria. Daptomycin, a lipopeptide, was also used in cardiac infectiology. Like all antibiotics, their use must be targeted to limit resistance (Figure 13) [43,44].

### 2.8. Quinolones

**Figure 14:**
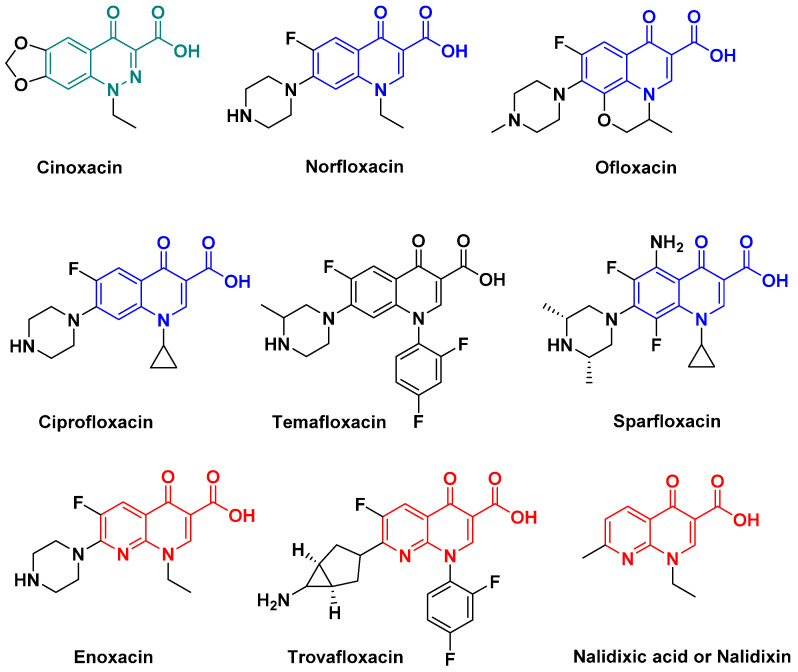
Examples of quinolones antibiotics (cinnoline, quinolone, and naphthyridine derivatives).

Quinolones are effective against various types of Gram-negative and Gram-positive bacteria [25]. They are bactericidal agents that inhibit the enzymatic function essential for DNA production and interfere with replication and transcription [45]. The first molecule was nalidixic acid initially envisaged as an antimalarial agent since it was identified during research on quinine. This class of antibiotics includes quinolones and naphthyridones, such as cinoxacin, norfloxacin, ofloxacin, ciprofloxacin, temafloxacin, sparfloxacin, nalidixic acid, enoxacin, etc. [46]; the list is obviously not exhaustive (Figure 14). Since their discovery in the 1960s, they have continued to evolve, with the aim of overcoming resistance or increasing their spectrum of activity or bioavailability (floxacin, ciprofloxacin, and levofloxacin). However, some compounds have been withdrawn from the market due to various side effects or toxicity (grepafloxacin, sparfloxacin, temafloxacin, trovafloxacin, Figure 14). Here again, quinolones and fluoroquinolones have been found in the environment in various countries, and are among the antibiotics most commonly detected outside so-called acceptable limits [47,48,49,50,51].

## 3. Main Natural Plant Sources Containing Compounds Claimed to Have Antibacterial Activity

Faced with the growing problem of resistance to the marketed antibiotics mentioned above, alternative treatments are currently being studied. However, it should be noted that few new synthetic structures are being studied. On the other hand, there is growing interest in the use of natural sources, whether of plant or marine origin. These natural molecules could be optimized to increase their efficacy while limiting resistance problems. Various studies on the phenomenon of resistance have shown that it results either from genetic modifications or from misuse and/or medical recommendations [52,53,54,55,56,57,58,59,60]. As natural compounds are not infinitely available, analytical chemistry research is also needed to optimize their extraction. New efficient synthesis routes will also be required to limit the overexploitation of resources.

In this study, we will focus on antibacterials commonly found in foods, spices, and beverages such as tea and herbal teas. Among the best botanical sources of antibacterials are garlic (*Allium sativum*), ginger (*Zingiber officinale*), oregano (*Origanum vulgare*), thyme (*Thymus vulgaris*), clove (*Syzygium aromaticum*), and ravintsara (*Cinnamomum camphora*), consumed as a tea or essential oil. Andrographis *(Andrographis paniculata)* and echinacea (*Echinacea purpura*) are consumed mainly as a dietary supplement. Burdock (*Arctium lappa)* is consumed as a vegetable or tea, lapacho tea (*Handroanthus impetiginosus*), green tea (*camellia sinensis*), and turmeric (*Curcuma longa*). Some are known to reduce toxin production by *B. cereus* and *Clostridioides* [61,62]. Added to this is chitosan, derived from the chitin in the shells of marine organisms (crustaceans) and the subject of much research for its antibiofilm activity. This property is of particular interest when it comes to fighting resistance phenomena. The compounds contained in these natural plant sources are claimed to destabilize bacterial cell architecture, inducing increased permeability. This variation in permeability would disrupt various vital cellular activities and processes, and these compounds would also inhibit the efflux pumps involved in antibiotic resistance mechanisms [63,64,65]. Similarly, as these natural sources are made up of a mixture of molecules with lipophilic and hydrophobic properties, in addition to their action on the bacterial cell membrane, they would have an impact on membrane fatty acids. Their impact is also claimed depending on cell morphology [66,67]. It is problematic to establish activity tables for these compounds, as the literature reports biological tests on various bacterial strains carried out under non-analogous conditions. It would be necessary to carry out a large-scale study on different plant sources with constant parameters. However, there is a structural relationship between the components of plant sources and antibacterial activity, as well as synergistic interactions. For example, in the case of terpenoids present in various plant sources, antimicrobial activity has been shown to be linked to the chemical functions they contain. Hydroxyl groups and the presence of delocalized electrons in phenolic terpenoids have an important function against microorganisms [68]. However, although antimicrobial activities are claimed and well established, these plant sources remain less effective than synthetic or semisynthetic compounds. Interestingly, they have a synergistic effect with antibiotic drugs and could be used as a multi-target therapeutic strategy for the treatment of antibiotic-resistant bacterial infectious diseases [69]. Indeed, because their mode of action affects several targets at once, this makes it difficult to establish bacterial adaptation and hence specific resistance [70]. These natural products can either improve the penetration of antibiotics through bacterial membranes or hinder efflux pumps, which represents a considerable improvement. The choice and order in which the plant sources mentioned in this review are discussed was made because of the importance of their claims as natural antibiotics in the literature.

### 3.1. Garlic

**Figure 15:**
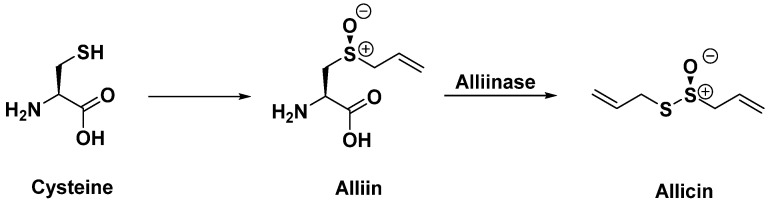
Enzymatic transformation of alliin.

Garlic contains phenolic acids, which give it antiseptic properties, as well as various flavonoids. The most important compound is alliin, which, under the action of an enzyme called alliinase, transforms into allicin, a compound with antibacterial properties that has shown very promising results on penicillin-resistant strains of *S. aureus*. However, allicin is not very stable and oxidizes rapidly, leading to compounds that also appear to play a role in therapeutic effects [71,72] (Figure 15).

### 3.2. Ginger

**Figure 16:**
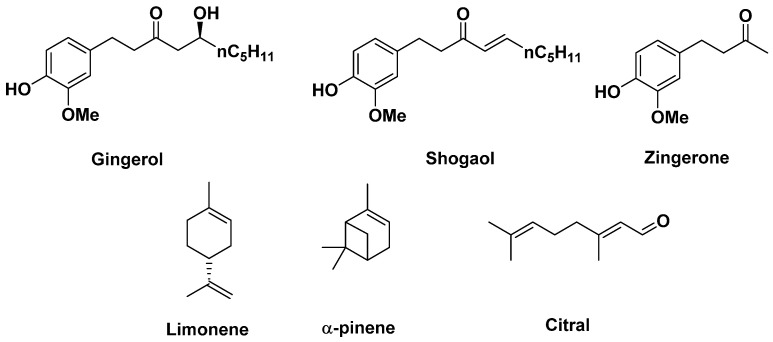
Some compounds found in ginger.

Ginger contains gingerol, zingerone [73], shogaol [74], and citral, which are recognized for their antioxidant and anti-inflammatory activities. The molecules present in this root that are thought to be responsible for its antimicrobial activity are limonene and α-pinene (Figure 16). Limonene is thought to inhibit bacterial proliferation, in particular Gram-positive *E. coli*, with an action on the bacterial membrane leading to cell death, while α-pinene is a bacteriostatic agent that merely inhibits bacterial growth, but again only on Gram-positive bacteria, making this root a restricted-spectrum antibiotic [73,74].

### 3.3. Oregano

**Figure 17:**
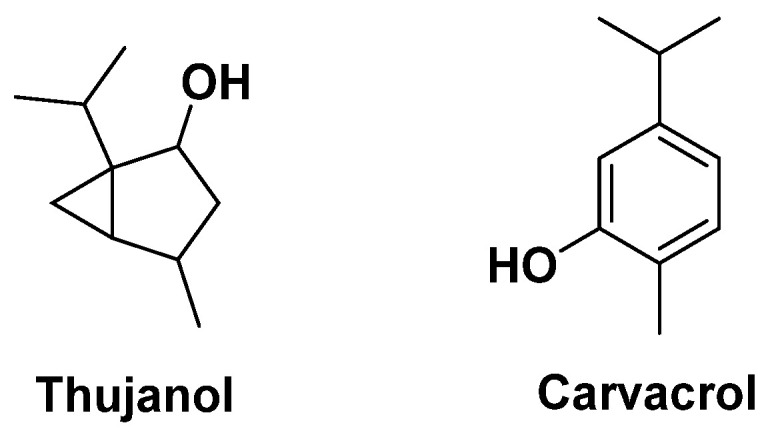
Basic compounds found in oregano with antimicrobial activity.

There are two main families: marjoram (*Origanum majorana*), rich in thujanol (mono-terpenol, Figure 17), and oregano (*Origanum vulgare*), rich in carvacrol. The former is an effective antimicrobial and antiviral agent, while the latter has antibacterial properties. The latter are effective against *H. pylori* and could also be effective against the *Staphylococci* involved in cystic fibrosis, which have the capacity to develop resistance to the usual antibiotics [75,76].

### 3.4. Thyme

**Figure 18:**
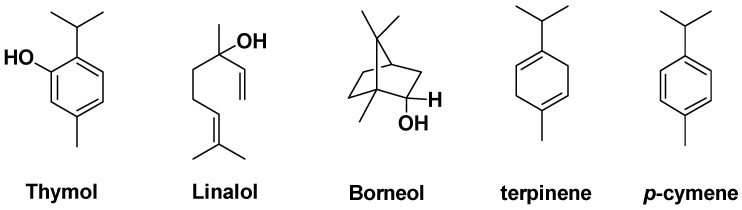
Basic compounds found in thyme with antimicrobial activity.

The most interesting and abundant constituents of thyme are monoterpene hydrocarbon compounds, which are the biogenetic precursors of thymol, *p*-cymene, and carvacrol, not to mention the presence of a number of flavonoids. Like oregano, these compounds have antiseptic activity against *H. pylori* and antifungal properties. Thymol is used for its numerous biological and pharmacological properties: anti-tumor, antioxidant, anti-inflammatory [77] and to fight certain oral bacteria that cause unpleasant odors [78]. Gallucci et al. have reported that thymol reduces bacterial resistance to certain antibiotics such as penicillin [79]. In addition to thymol, the flavones contained in the plant also appear to contribute to its biological properties [80]. The antibacterial action of thymol causes either disruption of the cytoplasmic membrane, or alteration of enzymes involved in ATP synthesis [68], or in the composition of *E. coli* constituents [81]. An increase in cell permeability (*P. aeruginosa and S. aureus*) is also claimed [82]. Carvacrol, with its multiple therapeutic properties [83,84], is a monoterpenoid phenol found in a variety of plants, all referenced as potential antibacterial agents, such as thyme, oregano, and wild bergamot, used as a food or cosmetic additive for its antimicrobial activity, notably against *E. coli*, *Salmonella*, and *B. cereus* [85]. It is claimed to act primarily on bacterial membranes [86,87,88]. *P*-cymene, a natural aromatic hydrocarbon, is the biological precursor of carvacrol and thymol. It has only a weak antibacterial effect, but is thought to enhance the effect of thymol and/or carvacrol [87] (Figure 17) and to act in conjunction with linalol, terpinene, and borneol [89] (Figure 18).

In terms of structural relationships between components and antibacterial activity, it has been shown that in phenolic terpenoids, hydroxyl groups and the presence of delocalized electrons play an important role in antibacterial activity. The importance of the hydroxyl group in the phenolic structure was also confirmed. Indeed, if the hydroxyl group of carvacrol is methylated, the change in its hydrophobicity affects activity. The same applies to the relative position of the hydroxyl group, as demonstrated by the difference in activity between carvacrol and thymol against Gram-negative and Gram-positive bacteria. Similarly, the presence of an acetate seems to improve activity when comparing geraniol and geranyl acetate, or borneol and bornyl acetate. The presence of α/β unsaturated carbonyls, ketones or aldehydes seems to contribute to activity [68,69,70,71,72,73,74,75,76,77,78,79,80,81,82,83,84,85,86,87,88,89,90].

### 3.5. Cloves

**Figure 19:**
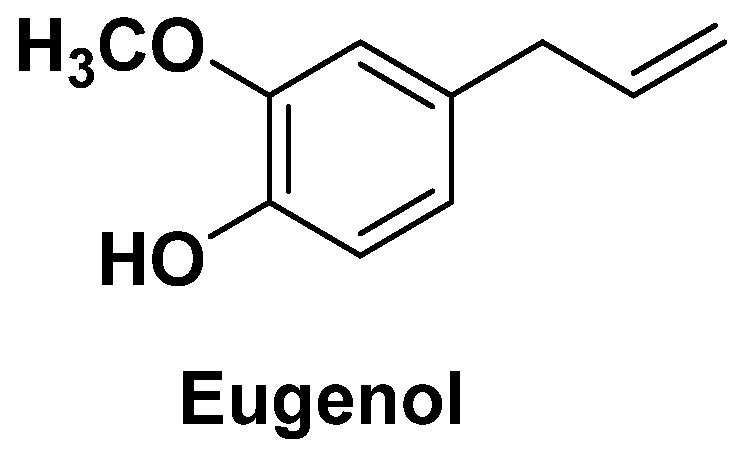
Eugenol or 4-allyl-2-methoxyphenol.

Cloves contain eugenol or 4-allyl-2-methoxyphenol (Figure 19), a bactericidal, analgesic, antiseptic, and anesthetic compound found in various therapeutic formulations, mainly used in dentistry, but also in cosmetics for its aromatic properties. Eugenol is also present in many other plants, such as cinnamon cloves, bay leaves, and certain peppers. In addition to its antioxidant and anti-inflammatory activities, eugenol has also demonstrated excellent broad-spectrum antifungal and antimicrobial activity against many Gram-negative and Gram-positive bacteria. Clove has very interesting effects on multi-resistant microorganisms. It could therefore play a role in the development of future antibiotics [91,92,93].

### 3.6. Ravintsara

**Figure 20:**
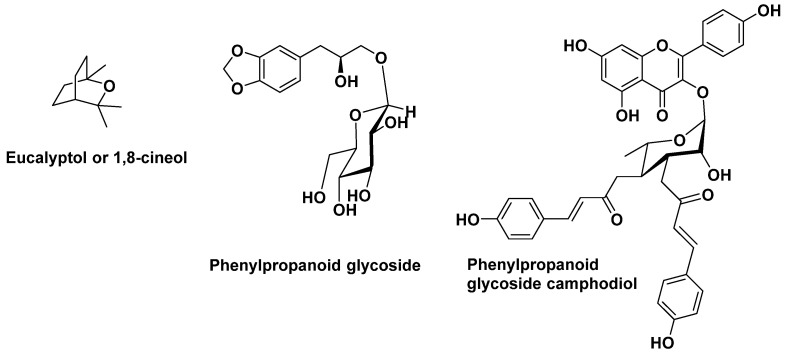
Some constituents found from the leaves of *Cinnamon camphora cineole* [96].

A study was carried out on the essential oil of *Cinnamomum camphora cineole* or ravintsara (Figure 20), reputed to be immunostimulant, antibacterial, and without risk of toxicity [94]. The aim of this study was to find natural molecules that could limit nosocomial infections encountered in hospitals, mainly in operating theatres and especially in intensive care units [95] Ravintsara is a tree about 3 to 5 m high when cultivated, and belongs to the *Lauraceae* family. The leaves are used, either as a decoction or as an essential oil composed mainly of 1,8-cineole or eucalyptol (55%), terpenes, and alphaterpineol (8%), a monoterpene alcohol. If not immunostimulant, these derivatives are at least immunomodulating. In this hospital study, diffusion of this essential oil, at a rate of 10 cc of ravintsara per day and per room (approximately 70 m^3^ of volume every two hours), significantly reduced nosocomial pneumopathies in the wards concerned. According to the authors, the high cost of this essential oil appears to be more than offset by its efficacy and the significant reduction in hospitalization days.

### 3.7. Andrographis and Echinacea

**Figure 21:**
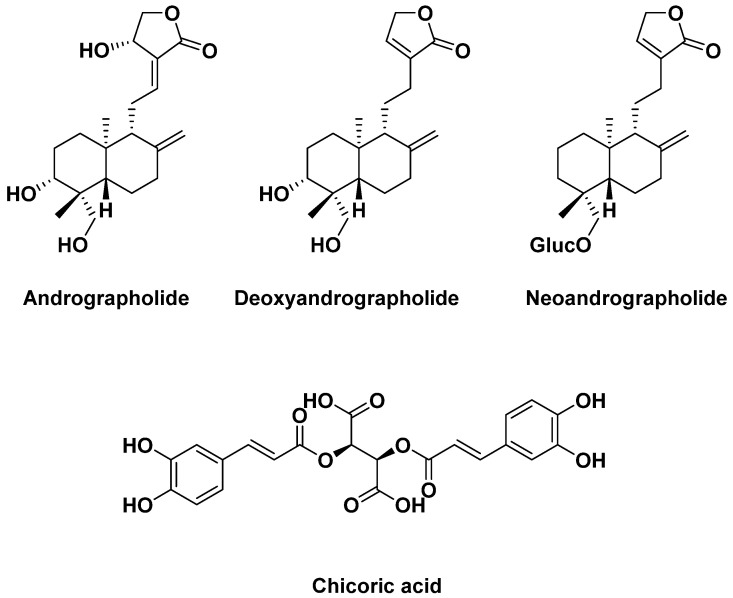
Compounds structures contained in andrographis and echinacea (chicoric acid).

One of the main components of *andrographis paniculata* is andrographolide, widely recognized for its anti-inflammatory properties [97,98,99,100,101]. This natural molecule has been chemically pharmacomodulated by numerous researchers to optimize its anticancer properties [102]. Andrographolide is the most important ingredient but other compounds such as deoxyandrographolide and neoandrographolide are also present in large quantities (Figure 21). This plant, marketed in the form of dried leaves and stems, is used as a dietary supplement for respiratory pathologies, in capsule or tablet form. G. Gancitano et al. [103] showed, in a very recent study of numerous clinical trials, that the use of *echinacea* also significantly reduces the need for antibiotic therapy in respiratory tract disorders, making it possible to envisage interesting synergies. One of the main components of *echinacea* is chicoric acid (Figure 21)

### 3.8. Burdock

**Figure 22:**
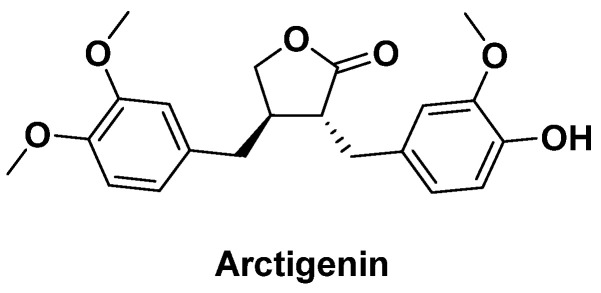
Arctigenin compound with antibacterial activity.

The root of *Arctium lappa* (burdock) contains a high proportion of the polyphenolic compound arctigenin (Figure 22), whose antibacterial activity against *P. aeruginosa* was recently assessed by A. E. Koshak et al. [104]. They measured its effect on the bacterial cell membrane and showed that antibacterial activity was due to its ability to disrupt the cell membrane by modifying the production of extracellular enzymes. In addition, they were able to show that this natural compound, like those already mentioned, could have a synergistic effect with various antibiotics and did not appear to induce resistance, hence its interest as a complementary agent in the treatment of *P. aeruginosa*-induced infections.

### 3.9. Lapacho

**Figure 23:**
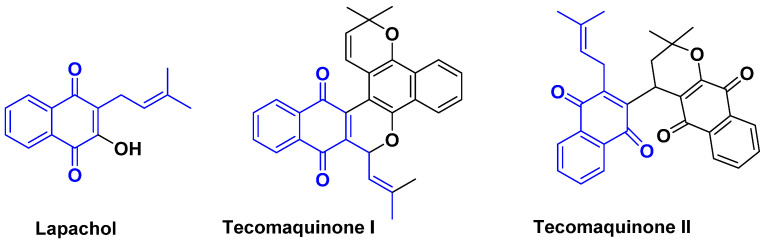
Lapachol and tecomaquinine I and II structures.

*Lapacho bark* is empirically known for its antibacterial properties. It is known to contain several quinones, including vitamin K and quinine. Natural quinones as a whole are attracting the attention of scientists for their antibiotic and anticancer properties. Some are well known for their antioxidant action and their ability to penetrate cell membranes, particularly cancer cells, to destroy them. Among the quinones present in *Lapacho bark* are the interesting tecomaquinones I and II (Figure 23) [105]. Lapachol and xyloidin are recognized and referenced as highly effective antibiotics, antivirals, and anti-inflammatories. Their strong antibacterial, antiparasitic, and antifungal activities on simple contact make them an effective natural source against *H. pylori*, *S. aureus*, which are known to develop resistance to conventional antibiotics and certain skin infections.

### 3.10. Camellia Sinensis, Tea Tree

Epigallocatechin gallate (EGCG), known for its anti-inflammatory and antioxidant effects [88,94], could prove very useful against bacterial growth and in the fight against bacterial resistance, as mentioned in recent studies [106,107].

ECGC could be used in water treatment to reduce microbial pollution [106] without toxicity problems. Siriphan et al. [107] recently reported that ECGC had bactericidal activity on MDR strains of *V. cholerae* and that its use in combination with tetracycline increased the response compared with either product used in isolation.

**Figure 24:**
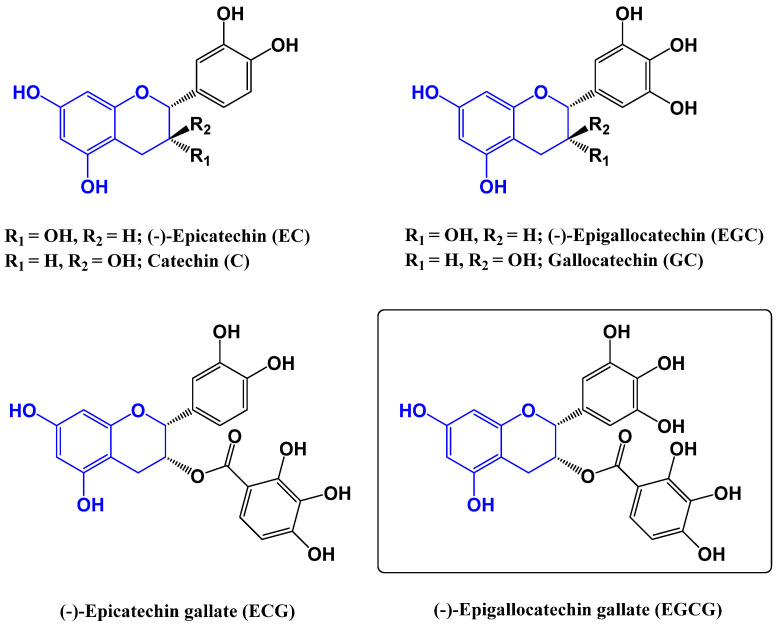
Main compound found in *Camellia sinensis* (tea tree) with multiple activities.

EGCG could also restore the activity of aztreonam, an antibiotic used to fight *P. aeruginosa*, a strain which is developing increasing resistance, hence the interest in this combination [108]. Here again, EGCG and aztreonam appear to have a synergistic effect. This synergistic effect is thought to derive from EGCG’s ability to increase bacterial membrane permeability and thus promote antibiotic absorption (Figure 24).

### 3.11. Curcuma

**Figure 25:**
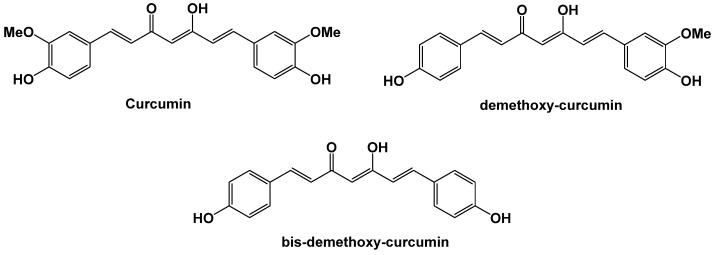
Various curcumin derivatives present in *Curcuma longa* with multiple activities.

The rhizome of *Curcuma longa*, which contains curcumin (Figure 25), is one of the natural bioactive substances that has been the subject of numerous studies in recent years. Curcumin has also been pharmacomodulated to optimize its anti-inflammatory and antioxidant properties [100]. These biological properties are well documented, and curcumin has also shown promising interest in the treatment of infections, with both bacterial and antibacterial activities [109]. Like some of the above-mentioned natural substances, curcumin in combination with some antibiotics can increase the sensitivity of bacteria to antibiotics in vitro [110]. Its use in combination with several antibiotics has been shown to have a synergistic effect [111,112]. Further studies are needed to determine whether curcumin alone has antibacterial properties.

### 3.12. Chitosan

Bacteria are characterized by quorum sensing (QS), the set of molecules that bacteria use to coordinate communication between themselves. It regulates a number of cellular functions, including the formation of biofilms, the extracellular matrix that covers and protects the community [113]. QS is therefore associated with the development of antibiotic resistance. Molecules that could have an anti-QS effect [114] and inhibit biofilm formation would also be of great interest in resistance phenomena. For example, *S. aureus*, which is often resistant to antibiotics, uses biofilm as a key virulence factor for its persistence. The biofilms of many bacteria therefore represent a major challenge in the fight against bacterial infections. Among the molecules of marine origin is chitosan [115]. This molecule is extracted from crustacean shells and studied by numerous research teams for its multiple biological activities, including the anti-biofilm activities of interest here. This molecule is a polymer of (β-1/4)-2-amino-2-deoxy-D-glucopyranose and can have different molecular weights depending on the number of associated glycosidic units. Biological activity depends on these different forms. To increase its solubility in water, various chemical modifications have been considered [116]. Chitosan is produced commercially from chitin. The degree of deacetylation determines its biological applications [116]. Chitosan induces cell wall rupture and altered membrane permeability, followed by inhibition of DNA replication, leading to cell death. It can also inhibit microbial growth [117]. Chitosan comes in a variety of forms and Usman et al. [118] have shown that it can be used in the form of nanoparticles (NPs). These Cu-Chitosan-NPs showed antifungal and antibacterial activity against several bacteria: MRSA, *S. choleraesuis*, *B. subtilis*, *P. aeruginosa*, or *C. albicans*.

Recently, O. AL-Fawares et al. [119] also reported very interesting results from chitosan nanoparticles obtained by 95% deacetylation of chitin. They achieved inhibition of biofilm formation for *C. jejuni*, *P. aeruginosa*, and *E. coli* treated with these NPs.

## 4. Conclusions

These various natural products, whose list is not exhaustive, have obvious antibacterial properties, often coupled with anti-fungal, anti-inflammatory, and antiseptic properties. Some of the constituents of these plants may have structural analogies with classic antibiotics, but their activities appear to be due to synergistic effects. Used alone, these plants are of course not as effective as marketed antibiotics. However, they are capable of increasing antibiotic efficacy by facilitating membrane permeability. They also play a role in efflux pumps helping to limit resistance. The structures are sometimes simple but act in synergy with the numerous flavonoids present in these plants. Indeed, the constituents mentioned here, which are present in greater quantities, may not be the most active molecules in these plants or medicinal herbs. Most studies in the literature are carried out on essential oils or decoctions, and not on isolated compounds. They therefore represent a source of inspiration for the research and development of new antibiotics. New, effective, multi-component therapies can also be envisaged to solve the huge public health problem of antibiotic resistance. Over-consumption of antibiotics, like that of other drugs, presents us with major challenges, whereas a carefully selected human or animal diet could enable us to significantly limit our growing need for drugs.

## References

[1] Bottalico L., Charitos I.A., Potenza M.A., Montagnani M., Santacroce L. (2022). The war against bacteria, from the past to present and beyond. Expert Rev. Anti-Infect. Ther..

[2] Colomb-Cotinat M., Lacoste J., Brun-Buisson C., Jarlier V., Coignard B., Vaux S. (2016). Estimating the morbidity and mortality associated with infections due to multidrug-resistant bacteria (MDRB), France, 2012. Antimicrob. Resist. Infect. Control.

[3] Van Boeckel T.P., Brower C., Gilbert M., Grenfell B.T., Levin S.A., Robinson T.P., Teillant A., Laxminarayan R. (2015). Global trends in antimicrobial use in food animals. Proc. Natl. Acad. Sci. USA.

[4] Alain L.F.A. (2017). Antibiotics and Antibiotic Resistance. Biomed. J. Sci. Tech. Res..

[5] Muteeb G., Rehman M.T., Shahwan M., Aatif M. (2023). Origin of Antibiotics and Antibiotic Resistance, and Their Impacts on Drug Development: A Narrative Review. Pharmaceuticals.

[6] Ventola C.L. (2015). The antibiotic resistance crisis: Part 1: Causes and threats. Pharm. Ther..

[7] Antimicrobial Resistance. https://www.who.int/news-room/fact-sheets/detail/antimicrobial-resistance.

[8] Lieberman J.M. (2023). Appropriate antibiotic use and why it is important: The challenges of bacterial resistance. Pediatr. Infect. Dis. J..

[9] Guedes B.N., Krambeck K., Durazzo A., Lucarini M., Santini A., Oliveira M.B.P.P., Fathi F., Souto E.B. (2024). Natural antibiotics against antimicrobial resistance: Sources and bioinspired delivery systems. Braz. J. Microbiol..

[10] Serreau R., Amirouche A., Benyamina A., Berteina-Raboin S. (2024). Propranolol Hydrochloride Psychiatric Effectiveness and Oxidative Stress: An Update. Oxygen.

[11] Goldmann D.A., Weinstein R.A., Wenzel R.P., Tablan O.C., Duma R.J., Gaynes R.P., Schlosser J., Martone W.J. (1996). Strate-gies to Prevent and Control the Emergence and Spread of Antimicrobial-Resistant Microorganisms in Hospitals. A challenge to hospital leadership. JAMA.

[12] Kollef M.H., Fraser V.J. (2001). Antibiotic Resistance in the Intensive Care Unit: Strategies for Management. Ann. Intern. Med..

[13] Szczepanowski R., Linke B., Krahn I., Gartemann K.H., Gutzkow T., Eichler W., Puhler A., Schluter A. (2009). Detection of 140 clinically relevant antibiotic- resistance genes in the plasmid metagenome of wastewater treatment plant bacteria showing reduced susceptibility to selected antibiotics. Microbiology.

[14] Hulscher M.E.J.L., Grol R.P.T.M., van der Meer J.W.M. (2010). Antibiotic prescribing in hospitals: A social and behavioural scientific approach. Lancet Infect. Dis..

[15] Simoens S., De Corte N., Laekeman G. (2006). Clinical practice and costs of treating catheter-related infections with teicoplanin or vancomycin. Pharm. Pract..

[16] Moore L., Martin M., Quilici S. (2008). The cost-effectiveness of targeted prescribing of antimicrobials in Canada for community-acquired pneumonia in an era of antimicrobial resistance. Value Health.

[17] El-Shorbagi A.-N., Chaudhary S. (2019). Monobactams: A Unique Natural Scaffold of Four-Membered Ring Skeleton, Recent Development to Clinically Overcome Infections by Multidrug- Resistant Microbes. Lett. Drug Des. Discov..

[18] Papp-Wallace K., Endimiani A., Taracila M., Bonomo R. (2011). Carbapenems: Past, present, and future. Antimicrob. Agents Chemother..

[19] Brown A.G., Butterworth D., Cole M., Hanscomb G., Hood J.D., Reading C., Rolinson G.N. (1976). Naturally-occurring beta-lactamase inhibitors with antibacterial activity. J. Antibiot..

[20] Etebu E., Arikekpar I. (2016). Antibiotics: Classification and mechanisms of action with emphasis on molecular perspectives. Int. J. Appl. Microbiol. Biotecnol. Res..

[21] Overbye K.M., Barrett J.F. (2005). Antibiotics: Where did we go wrong?. Drug. Discov. Today..

[22] Torres J.A., Villegas M.V., Quinn J.P. (2007). Current concepts in antibiotic-resistant gram-negative bacteria. Expert Rev. Anti-Infect. Ther..

[23] Gobel A., Mc Ardell C.S., Joss A., Siegrist H., Giger W. (2007). Fate of sulfonamides, macrolides, and trimethoprim in different wastewater treatment technologies. Sci. Total Environ..

[24] Peterson L.R. (2008). Currently available antimicrobial agents and their potential for use as monotherapy. Clin. Microbiol. Infect..

[25] Mahajan G.B., Balachandran L. (2012). Antibacterial agents from actinomycetes—A review. Front. Biosci. (Elite Ed.).

[26] Talaro K.P., Chess B. (2008). Foundations in Microbiology.

[27] Donald G., Anderson M.D., Marjorie Jewell A.B. (1945). The Absorption, Excretion and Toxicity of Streptomycin in Man. N. Engl. J. Med..

[28] Löffler D., Ternes T.A. (2003). Analytical method for the determination of the aminoglycoside gentamicin in hospital wastewater via liquid chromatography-electrospray-tandem mass spectrometry. J. Chromatograph. A.

[29] Sanchez A.R., Rogers R.S., Sheridan P.J. (2004). Tetracycline and other tetracycline-derivative staining of the teeth and oral cavity. Int. J. Dermatol..

[30] Felman A., Begum F. (2023). Antibiotics: How Do Antibiotics Work? Medical News Today. https://www.medicalnewstoday.com/articles/10278.

[31] Chopra I., Roberts M. (2011). Tetracycline antibiotics: Mode of action, applications, molecular biology, and epidemiology of bacterial resistance. Microbiol. Mol. Biol. Rev..

[32] Fuoco D. (2012). Classification framework and chemical biology of tetracycline-structure-based drugs. Antibiotics.

[33] Giguère S., John F., Desmond J. (2006). Antimicrobial Therapy in Veterinary Medicine.

[34] Moore D. (2015). Antibiotic Classification and Mechanism. http://www.orthobullets.com/basic-science/9059/antibiotic-classification-and-mechanism.

[35] Lindberg R.H., Wennberg P., Johansson M.I., Tysklind M., Andersson B.A.V. (2005). Screening of human antibiotic substances and determination of weekly mass flows in five sewage treatment plants in Sweden. Environ. Sci. Technol..

[36] Skrzypczak N., Przybylski P. (2022). Modifications, biological origin and antibacterial activity of naphthalenoid ansamycins. Nat. Prod. Rep..

[37] Tenson T., Lovmar M., Ehrenberg M. (2003). The mechanism of action of macrolides, lincosamides and streptogramin B reveals the nascent peptide exit path in the ribosome. J. Mol. Biol..

[38] Bozdogan B., Appelbaum P.C. (2004). Oxazolidinones: Activity, mode of action, and mechanism of resistance. Int. J. Antimicrob. Agents.

[39] Kang H.-K., Park Y. (2015). Glycopeptide antibiotics: Structure and mechanism of action. J. Bacteriol. Virol..

[40] Yim G., Thaker M.N., Koteva K., Wright G. (2014). Glycopeptide antibiotic biosynthesis. J. Antibiot..

[41] Allen N.E., Nicas T.I. (2003). Mechanism of action of oritavancin and related glycopeptide antibiotics. FEMS Microbiol. Rev..

[42] Beauregard D.A., Williams D.H., Gwynn M.N., Knowles D.J.C. (1995). Dimerization and membrane anchors in extracellular targeting of vancomycin group antibiotics. Antimicrob. Agents Chemother..

[43] Jerala R. (2007). Synthetic lipopeptides: A novel class of anti-infectives. Expert Opin. Investig. Drugs.

[44] Kahne D., Leimkuhler C., Lu W., Walsh C. (2005). Glycopeptide and Lipoglycopeptide Antibiotics. Chem. Rev..

[45] Marzo A., Dal Bo L. (1998). Chromatography as an analytical tool for selected antibiotic classes: A reappraisal addressed to pharmacokinetic applications. J. Chromatogr. A.

[46] Domagala J.M. (1994). Structure-activity and structure-side-effect relationships for the quinolone antibacterials. J. Antimicrob. Chemother..

[47] Renuka K., Kapil A., Kabra S.K., Wig N., Das B.K., Prasad V.V.S.P., Chaudhry R., Seth P. (2004). Reduced susceptibility to ciprofloxacin and gyra gene mutation in north Indian strains of *Salmonella enterica* serotype Typhi and serotype Paratyphi A. Microb. Drug Resist..

[48] Ivanoff B., Levine M.M., Lambert P.H. (1994). Vaccination against typhoid fever: Present status. Bull. World Health Organ..

[49] Bhutta Z.A., Hendricks K.M. (1996). Nutritional management of persistent diarrhea in childhood: A perspective from the developing world. J. Pediatr. Gastroenterol. Nutr..

[50] Subekti D., Oyofo B.A., Tjaniadi P., Corwin A.L., Larasati W., Putri M., Simanjuntak C.H., Punjabi N.H., Taslim J., Setiawan B. (2001). *Shigella* spp. surveillance in Indonesia: The emergence or reemergence of *S. dysenteriae*. Emerg. Infect. Dis..

[51] Brooks J.T., Shapiro R.L., Kumar L., Wells J.G., Phillips-Howard P.A., Shi Y.-P., Vulule J.M., Hoekstra R.M., Mintz E., Slutsker L. (2003). Epidemiology of sporadic bloody diarrhea in rural western Kenya. Am. J. Trop. Med. Hyg..

[52] Wang Y.-Q., Li Q.-S., Zheng X.-Q., Lu J.-L., Liang Y.-R. (2021). Antiviral Effects of Green Tea EGCG and Its Potential Application against COVID-19. Molecules.

[53] Zhao J.-H., Wang Y.-W., Yang J., Tong Z.-J., Wu J.-Z., Wang Y.-B., Wang Q.-X., Li Q.-Q., Yu Y.-C., Leng X.-J. (2023). Natural Products as Potential Lead Compounds to Develop New Antiviral Drugs Over the Past Decade. Eur. J. Med. Chem..

[54] Bobate S., Mahalle S., Dafale N.A., Bajaj A. (2023). Emergence of environmental antibiotic resistance: Mechanism, monitoring and management. Environ. Adv..

[55] Lepe J.A., Martínez-Martínez L. (2022). Resistance mechanisms in Gram-negative bacteria. Med. Intensiv..

[56] Darby E.M., Trampari E., Siasat P., Gaya M.S., Alav I., Webber M.A., Blair J.M.A. (2023). Molecular mechanisms of antibiotic resistance revisited. Nat. Rev. Microbiol..

[57] Reygaert W.C. (2018). An overview of the antimicrobial resistance mechanisms of bacteria. AIMS Microbiol..

[58] Kresken M., Klare I., Wichelhaus T.A., Wohlfarth E., Layer-Nicolaou F., Neumann B., Werner G., Study Group ‘Antimicrobial Resistance’ of the Paul-Ehrlich-Society for Chemotherapy (2022). Glycopeptide resistance in *Enterococcus* spp. and coagulase-negative staphylococci from hospitalised patients in Germany: Occurrence, characteristics and dalbavancin susceptibility. J. Glob. Antimicrob. Resist..

[59] Urban-Chmiel R., Marek A., Stępień-Pyśniak D., Wieczorek K., Dec M., Nowaczek A., Osek J. (2022). Antibiotic Resistance in Bacteria—A Review. Antibiotics.

[60] Baig M.I.R., Kadu P., Bawane P., Nakhate K.T., Yele S., Ojha S., Goyal S.N. (2023). Mechanisms of emerging resistance associated with non-antibiotic antimicrobial agents: A state-of-the-art review. J. Antibiot..

[61] Mooyottu S., Kollanoor-Johny A., Flock G., Bouillaut L., Upadhyay A., Sonenshein A.L., Venkitanarayanan K. (2014). Carvacrol and trans-cinnamaldehyde reduce *Clostridium difficile* toxin production and cytotoxicity in vitro. Int. J. Mol. Sci..

[62] Seukep A.J., Kuete V., Nahar L., Sarker S.D., Guo M. (2020). Plant-derived secondary metabolites as the main source of efflux pump inhibitors and methods for identification. J. Pharm. Anal..

[63] Fadli M., Chevalier J., Bolla J.M., Mezrioui N.E., Hassani L., Pages J.M. (2012). Thymus maroccanus essential oil, a membranotropic compound active on Gram-negative bacteria and resistant isolates. J. Appl. Microbiol..

[64] Skandamis P.N., Nychas G.J. (2001). Effect of oregano essential oil on microbiological and physico-chemical attributes of minced meat stored in air and modified atmospheres. J. Appl. Microbiol..

[65] Oussalah M., Caillet S., Lacroix M. (2006). Mechanism of action of Spanish oregano, Chinese cinnamon, and savory essential oils against cell membranes and walls of *Escherichia coli* O157:H7 and Listeria monocytogenes. J. Food Prot..

[66] Sikkema J., de Bont J.A., Poolman B. (1995). Mechanisms of membrane toxicity of hydrocarbons. Microbiol. Rev..

[67] Heath R.J., Rock C.O. (2004). Fatty acid biosynthesis as a target for novel antibacterials. Curr. Opin. Investig. Drugs..

[68] Hyldgaard M., Mygind T., Meyer R.L. (2012). Essential oils in food preservation: Mode of action, synergies, and interactions with food matrix components. Front. Microbiol..

[69] Bush K., Courvalin P., Dantas G., Davies J., Eisenstein B., Huovinen P., Jacoby G.A., Kishony R., Kreiswirth B.N., Kutter E. (2011). Tackling antibiotic resistance. Nat. Rev. Microbiol..

[70] Langeveld W.T., Veldhuizen E.J., Burt S.A. (2014). Synergy between essential oil components and antibiotics: A review. Crit. Rev. Microbiol..

[71] Ankri S., Mirelman D. (1999). Antimicrobial properties of allicin from garlic. Microbes Infect..

[72] Cutler R.R., Wilson P. (2004). Antibacterial activity of a new, stable, aqueous extract of allicin against methicillin-resistant *Staphylococcus aureus*. Br. J. Biomed. Sci..

[73] Bitari B., Oualdi I., Touzani R., Elachouri M. (2022). *Zingiber officinale* Roscoe: A comprehensive review of clinical properties. Mater. Today Proc..

[74] Jolad S.D., Lantz R.C., Chen G.J., Bates R.B., Timmermann B.N. (2005). Commercially processed dry ginger (*Zingiber officinale*): Composition and effects on LPS-stimulated PGE_2_ production. Phytochemistry.

[75] Spagnolettia A., Guerrinia A., Tacchinia M., Vinciguerrab V., Leonec C., Marescaa I., Simonettic G., Sacchettia G., Angiolellac L. (2016). Chemical Composition and Bio-efficacy of Essential Oils from Italian Aromatic Plants: *Mentha suaveolens*, *Coridothymus capitatus*, *Origanum hirtum* and *Rosmarinus officinalis*. Nat. Prod. Commun..

[76] Carrasco A., Perez E., Cutillas A.-B., Martinez-Gutierrez R., Tomas V., Tudela J. (2016). *Origanum vulgare* and *Thymbra capitata* Essential Oils from Spain: Determination of Aromatic Profile and Bioactivities. Nat. Prod. Commun..

[77] Bhalla Y., Gupta V.K., Jaitak V. (2013). Anticancer activity of essential oils: A review. J. Sci. Food Agric..

[78] Botelho M.A., Nogueira N.A.P., Bastos G.M., Fonseca S.G.C., Lemos T.L.G., Matos F.J.A., Montenegro D., Heukelbach J., Rao V.S., Brito G.A.C. (2007). Antimicrobial activity of the essential oil from *Lippia sidoides*, carvacrol and thymol against oral pathogens. Braz. J. Med. Biol. Res..

[79] Gallucci M.N., Oliva M., Casero C., Dambolena J., Luna A., Zygadlo J., Demo M. (2009). Antimicrobial combined action of terpenes against the food-borne microorganisms *Escherichia coli*, *Staphylococcus aureus* and *Bacillus cereus*. Flavour. Frag. J..

[80] Keyhanmanesh R., Boskabady M.H. (2012). Relaxant effects of different fractions from *Tymus vulgaris* on guinea-pig tracheal chains. Biol. Res..

[81] Di Pasqua R., Mamone G., Ferranti P., Ercolini D., Mauriello G. (2010). Changes in the proteome of *Salmonella enterica* serovar Thompson as stress adaptation to sublethal concentrations of thymol. Proteomics.

[82] Lambert R.J.W., Skandamis P.N., Coote P.J., Nychas G.-J.E. (2001). A study of the minimum inhibitory concentration and mode of action of oregano essential oil, thymol and carvacrol. J. Appl. Microbiol..

[83] Suntres Z.E., Coccimiglio J., Alipour M. (2015). The Bioactivity and Toxicological Actions of Carvacrol. Crit. Rev. Food Sci. Nutr..

[84] Fachini-Queiroz F.C., Kummer R., Estevão-Silva C.F., Carvalho M.D.D.B., Cunha J.M., Grespan R., Bersanit-Amado C.A., Cuman R.K.N. (2012). Effects of Thymol and Carvacrol, Constituents of *Thymus vulgaris* L. Essential Oil, on the Inflammatory Response. Evid.-Based Complement. Altern. Med..

[85] Oussalah M., Caillet S., Saucier L., Lacroix M. (2007). Inhibitory effects of selected plant essential oils on the growth of four pathogenic bacteria: *E. coli* O157:H7, *Salmonella typhimurium*, *Staphylococcus aureus* and Listeria monocytogenes. Food Control.

[86] Nostro A., Marino A., Blanco A.R., Cellini L., Di Giulio M., Pizzimenti F., Roccaro A.S., Bisignano G. (2009). In vitro activity of carvacrol against staphylococcal preformed biofilm by liquid and vapour contact. J. Med. Microbiol..

[87] Rudramurthy G.R., Swamy M.K., Sinniah U.R., Ghasemzadeh A. (2016). Nanoparticles: Alternatives Against Drug-Resistant Pathogenic Microbes. Molecules.

[88] Burt S. (2004). Essential oils: Their antibacterial properties and potential applications in foods—A review. Int. J. Food Microbiol..

[89] Cristani M., D’Arrigo M., Mandalari G., Castelli F., Sarpietro M.G., Micieli D., Venuti V., Bisignano G., Saija A., Trombetta D. (2007). Interaction of Four Monoterpenes Contained in Essential Oils with Model Membranes: Implications for Their Antibacterial Activity. J. Agric. Food Chem..

[90] Ultee A., Bennik M.H., Moezelaar R. (2002). The phenolic hydroxyl group of carvacrol is essential for action against the food-borne pathogen *Bacillus cereus*. Appl. Environ. Microbiol..

[91] Marchese A., Barbieri R., Coppo E., Orhan I.E., Daglia M., Nabavi S.F., Izadi M., Abdollahi M., Nabavi S.M., Ajami M. (2017). Antimicrobial activity of eugenol and essential oils containing eugenol: A mechanistic viewpoint. Crit. Rev. Microbiol..

[92] Hu Q., Zhou M., Wei S. (2018). Progress on the Antimicrobial Activity Research of Clove Oil and Eugenol in the Food Antisepsis Field. J. Food Sci..

[93] Purkait S., Bhattacharya A., Bag A., Chattopadhyay R.R. (2020). Synergistic antibacterial, antifungal and antioxidant efficacy of cinnamon and clove essential oils in combination. Arch. Microbiol..

[94] Blanchard J.M. (2007). Cinnamomum camphora à cinéole (ravintsara), une plante au service de la prévention des infections nosocomiales en milieu hospitalier. Phytothérapie.

[95] Gopanraj G., Dan M., Shiburaj S., Sethuraman M.G., George V. (2005). Chemical composition and antibacterial activity of the rhizome oil of *Hedychium larsenii*. Acta Pharm..

[96] Jiang X., Zhang X.-H., Li Y.-X., Chen K., Lin B., Lu T.-Q., Yang M., Chen G.-T., Fan B.-Y., Wang W.-L. (2023). Chemical constituents from the leaves of Cinnamomum camphora and their α-glucosidase inhibitory activities. Phytochem. Lett..

[97] Mussard E., Jousselin S., Cesaro A., Legrain B., Lespessailles E., Esteve E., Berteina-Raboin S., Toumi H. (2020). Andrographis paniculata and Its Bioactive Diterpenoids Protect Dermal Fibroblasts against Inflammation and Oxidative Stress. Antioxidants.

[98] Mussard E., Jousselin S., Cesaro A., Legrain B., Lespessailles E., Esteve E., Berteina-Raboin S., Toumi H. (2020). *Andrographis paniculata* and Its Bioactive Diterpenoids Against Inflammation and Oxidative Stress in Keratinocytes. Antioxidants.

[99] Villedieu-Percheron E., Ferreira V., Campos J.F., Destandau E., Pichon C., Berteina-Raboin S. (2019). Quantitative Determination of Andrographolide and Related Compounds in *Andrographis paniculata* Extracts and Biological Evaluation of Their Anti-Inflammatory Activity. Foods.

[100] Mussard E., Cesaro A., Lespessailles E., Legrain B., Berteina-Raboin S., Toumi H. (2019). Andrographolide, A Natural Antioxidant: An Update. Antioxidants.

[101] Messire G., Serreau R., Berteina-Raboin S. (2023). Antioxidant Effects of Catechins (EGCG), Andrographolide, and Curcuminoids Compounds for Skin Protection, Cosmetics, and Dermatological Uses: An Update. Antioxidants.

[102] Messire G., Rollin P., Gillaizeau I., Berteina-Raboin S. (2024). Synthetic Modifications of Andrographolide Targeting New Potential Anticancer Drug Candidates: A Comprehensive Overview. Molecules.

[103] Gancitano G., Mucci N., Stange R., Ogal M., Vimalanathan S., Sreya M., Booker A., Hadj-Cherif B., Albrich W.C., Woelkart-Ardjomand K. (2024). Echinacea Reduces Antibiotics by Preventing Respiratory Infections: A Meta-Analysis (ERA-PRIMA). Antibiotics.

[104] Koshak A.E., Elfaky M.A., Abdallah H.M., Albadawi D.A.I., Mohamed G.A., Ibrahim S.R.M., Alzain A.A., Khafagy E.-S., Rajab A.A.H., Hegazy W.A.H. (2024). Arctigenin from Burdock Root Exhibits Potent Antibacterial and Anti-Virulence Properties against *Pseudomonas aeruginosa*. J. Microbiol. Biotechnol..

[105] Kouidhi B., Zmantar T., Jrah H., Souiden Y., Chaieb K., Mahdouani K., Bakhrouf A. (2011). Antibacterial and resistance-modifying activities of thymoquinone against oral pathogens. Ann. Clin. Microbiol. Antimicrob..

[106] Zhang Y., Zhang Y., Ma R., Sun W., Ji Z. (2023). Antibacterial Activity of Epigallocatechin Gallate (EGCG) against *Shigella flexneri*. Int. J. Environ. Res. Public Health.

[107] Siriphap A., Kiddee A., Duangjai A., Yosboonruang A., Pook-In G., Saokaew S., Sutheinkul O., Rawangkan A. (2022). Antimicrobial Activity of the Green Tea Polyphenol (−)-Epigallocatechin-3-Gallate (EGCG) against Clinical Isolates of Multidrug-Resistant *Vibrio cholerae*. Antibiotics.

[108] Kanagaratnam R., Sheikh R., Alharbi F., Kwon D.H. (2017). An efflux pump (MexAB-OprM) of *Pseudomonas aeruginosa* is associated with antibacterial activity of Epigallocatechin-3-gallate (EGCG). Phytomedicine.

[109] Kali A., Bhuvaneshwar D., Charles P.M., Seetha K.S. (2016). Antibacterial synergy of curcumin with antibiotics against biofilm producing clinical bacterial isolates. J. Basic. Clin. Pharm..

[110] Karaman M., Firinci F., Ayyildiz Z.A., Bahar I.H. (2013). Effects of imipenem, tobramycin and curcumin on biofilm formation of *Pseudomonas aeruginosa* strains. Mikrobiyoloji Bul..

[111] Sasidharan N.K., Sreekala S.R., Jacob J., Nambisan B. (2014). In vitro synergistic effect of curcumin in combination with third generation cephalosporins against bacteria associated with infectious diarrhea. BioMed Res. Int..

[112] Teow S.Y., Ali S.A. (2015). Synergistic antibacterial activity of Curcumin with antibiotics against *Staphylococcus aureus*. Pak. J. Pharm. Sci..

[113] Trosko J.E. (2016). Evolution of Microbial Quorum Sensing to Human Global Quorum Sensing: An Insight into How Gap Junctional Intercellular Communication Might Be Linked to the Global Metabolic Disease Crisis. Biology.

[114] Szabó M.Á., Varga G.Z., Hohmann J., Schelz Z., Szegedi E., Amaral L. (2010). Inhibition of quorum-sensing signals by essential oils. Phytother. Res..

[115] Zhang S., Ji Y., He Y., Dong J., Li H., Yu S. (2024). Effect of Environmental pH on the Mechanics of Chitin and Chitosan: A Single-Molecule Study. Polymers.

[116] Hosseinnejad M., Jafari S.M. (2016). Evaluation of different factors affecting antimicrobial properties of chitosan. Int. J. Biol. Macromol..

[117] Divya K., Shanavas J. (2018). Chitosan nanoparticles preparation and applications. Environ. Chem. Lett..

[118] Usman M.S., El Zowalaty M.E., Shameli K., Zainuddin N., Salama M., Ibrahim N.A. (2013). Synthesis, characterization, and antimicrobial properties of copper nanoparticles. Int. J. Nanomed..

[119] Al-Fawares O., Alshweiat A., Al-Khresieh R.O., Alzarieni K.Z., Rashaid A.H.B. (2024). A significant antibiofilm and antimicrobial activity of chitosan-polyacrylic acid nanoparticles against pathogenic bacteria. Saudi Pharm. J..

